# Differences in Clinicopathological Characteristics of Papillary Thyroid Carcinoma between Symptomatic and Asymptomatic Patients with Primary Hyperparathyroidism

**DOI:** 10.1155/2021/9917694

**Published:** 2021-05-31

**Authors:** Yuan Liu, Siyi Guo, Shaowei Sang, Jinbo Liu, Lin Qi, Bin Lv, Xiaoli Zhang

**Affiliations:** ^1^Department of Endocrinology, Qilu Hospital of Shandong University, Jinan 250012, China; ^2^Institute of Endocrine and Metabolic Diseases of Shandong University, Jinan 250012, China; ^3^Clinical Epidemiology Unit, Qilu Hospital of Shandong University, Jinan 250012, China; ^4^Department of Thyroid Surgery, General Surgery, Qilu Hospital of Shandong University, Jinan 250012, China

## Abstract

**Background:**

Popularization of cervical ultrasound led to higher detection of papillary thyroid carcinoma (PTC) and primary hyperparathyroidism (PHPT), as well as increasing percentage of asymptomatic PHPT in China. Although the coexistence of PTC and PHPT has been reported, it is unknown whether the clinicopathological features of PTC differ between asymptomatic and symptomatic PHPT patients.

**Methods:**

We retrospectively reviewed the medical records of 304 PHPT patients treated in our hospital between January 2009 and July 2020, including 217 females and 87 males with the average age of 53.27 ± 13.54 years. Of the 304 patients, 181 were symptomatic PHPT patients and 123 were asymptomatic PHPT patients. We analyzed the laboratory results, postoperative pathology, and the TNM stage of PTC between asymptomatic and symptomatic PHPT patients.

**Results:**

Concurrent thyroid nodules were found in 61.51% of PHPT patients, and the prevalence of PTC in thyroidectomized PHPT patients was 29.89% in our cohort. Lower serum parathyroid hormone (PTH) (*p* < 0.05) and calcium (*p* < 0.05) were found in PHPT patients with PTC compared to patients with benign thyroid lesion. Compared with the symptomatic PHPT patients, the asymptomatic PHPT patients showed lower serum calcium (*p* < 0.05), serum chlorine (*p* < 0.05), alkaline phosphatase (*p* < 0.05), PTH (*p* < 0.05), and bone turnover markers (*p* < 0.05) but higher prevalence of thyroid nodules (70.73% versus 55.24%, *p* < 0.05) and PTC (15.44% versus 3.87%, *p* < 0.05). All the PTC in symptomatic PHPT patients were papillary microcarcinoma limited to the thyroid, while 68.42% and 15.78% of the PTC in asymptomatic PHPT patients showed microscopic extrathyroidal extension and lymph node metastases, respectively. Moreover, 36.84% (7/19) of the PTC patients in asymptomatic group showed multifocality, which was much higher than 14.29% (1/7) in the symptomatic group; however, no statistical significance was found (*p*=0.24).

**Conclusions:**

The concomitant PTC in asymptomatic PHPT patients showed a higher rate of microscopic extrathyroidal invasion when compared to symptomatic PHPT patients. So the FNA is essential to the asymptomatic PHPT patients with suspicious thyroid nodules and once the PTC is confirmed, concurrent parathyroidectomy should be performed with thyroidectomy in asymptomatic PHPT patients.

## 1. Introduction

Primary hyperparathyroidism (PHPT) is the third most common endocrine and metabolic disease with an incidence of 3/1000 [1]. Due to the popularization of serum calcium measurements and neck ultrasound, the clinical spectrum of PHPT in China has been changing from predominance of symptomatic PHPT to asymptomatic PHPT [2, 3]. Likewise, the rapid increase of thyroid carcinoma is also closely related to the higher availability of cervical ultrasound. Among the histological types, papillary thyroid carcinoma (PTC) is the most common one, accounting for 95.1%–96.1% of all thyroid malignancies [4, 5]. Previous studies proved that the cancer behaviour was various in different population. For example, a higher rate of central lymph node metastasis was observed in PTC patients with positive HBsAg [6], and higher invasive ability and multifocality of PTC were found in the PHPT patients [7]. However, it remains unclear whether there are differences in the incidence, the tumor size, and the invasive ability of PTC between asymptomatic and symptomatic PHPT patients. In this study, we investigated the differences in the clinicopathological characteristics of PTC between asymptomatic and symptomatic PHPT patients.

## 2. Patients and Methods

We retrospectively reviewed medical records of the PHPT patients admitted to our hospital between January 2009 and July 2020. The data from the electronic health records was abstracted including the present histories, family histories, laboratory results, imaging examinations, and the pathological examinations. No informed consent was required from the patients for the present study because we only retrospectively accessed a deidentified database for analysis purposes. This study was approved by the Clinical Research Ethics Committee of Qilu Hospital, Shandong University.

The diagnosis of PHPT was established by the presence of hypercalcemia and concomitant aberrantly elevated serum parathyroid hormone (PTH) level or inappropriately normal PTH level; to be specific, the PTH was higher than 20 pg/ml when the serum calcium was above 2.6 mmol/l [8]. The diagnosis of asymptomatic PHPT was based on the absence of typical symptoms or signs related to hypercalcemia and diagnosed accidentally by the serum calcium examination or neck ultrasound. The symptomatic PHPT patients were admitted mainly for parathyroidectomy and treatment of hypercalcemia. The asymptomatic PHPT patients in our study were hospitalized in the following situation: (1) the patients who met the criteria of parathyroidectomy (*N* = 105), (2) patients with a history of asymptomatic PHPT who were hospitalized because of concomitant diseases (*N* = 2), and (3) newly diagnosed asymptomatic PHPT patients by calcium examination during the routine inpatient examination when admitted for concomitant diseases (*N* = 16). The concomitant diseases included diabetes mellitus (*N* = 6), hypertension (*N* = 4), leukemia (*N* = 1), gastric cancer (*N* = 2), ischemic stroke (*N* = 2), infection (*N* = 2), and nephrotic syndrome (*N* = 1). Patients with secondary or tertiary hyperparathyroidism or multiple endocrine neoplasia were excluded.

The laboratory results included serum calcium (reference range, 2.00–2.60 mmol/L), phosphate (0.60–1.60 mmol/L), magnesium (0.65–1.1 mmol/L), potassium (3.25–5.3 mmol/L), sodium (137–147 mmol/L), chlorine (99–110 mmol/L), alkaline phosphatase (ALP) (50–135 U/L), albumin (40–55 g/L), PTH (15–65 pg/mL; Roche Diagnostics GmbH, Germany), 25-hydroxyvitamin D (25 (OH)D) (≥30 ng/mL; Roche Diagnostics GmbH, Germany), N-bone Gla-protein (N-BGP) (premenopausal: 14–43 ng/mL, postmenopausal: 15–46 ng/mL; Roche Diagnostics GmbH, Germany), *β*-isomerized C-terminal telopeptides (*β*-CTx) (premenopausal: ≤0.57 ng/mL, postmenopausal: ≤1.008 ng/mL; Roche Diagnostics GmbH, Germany), and total procollagen type 1 amino-terminal propeptide (TP1NP) (premenopausal: 15.13–58.59 ng/mL, postmenopausal: 20.25–76.31 ng/mL; Roche Diagnostics GmbH, Germany). The albumin-corrected serum calcium levels (mmol/L) were calculated using the following formula: serum calcium concentration = measured serum calcium (mmol/L) + (40 − serum albumin (g/L)) × 0.02.

The thyroid nodule was confirmed by ultrasound examination, and the Thyroid Imaging Reporting and Data System (TIRADS) was used to evaluate the malignant risk of thyroid nodules [9]. Fine needle aspiration (FNA) has been carried out in our hospital since July 2016. Nodules with the highly suspicious pattern underwent diagnostic FNA when its diameter was bigger than 1 cm and cytology was used to refute or confirm malignancy. The thyroid pathological entities, including nodular goiter, carcinoma, lymphocytic thyroiditis, and thyroid adenoma, were confirmed by pathohistological examination after surgery; and the pathological characteristics of thyroid carcinoma including the multifocality, microscopic extrathyroidal invasion, and lymph node metastases were confirmed by the same experienced pathologist.

All statistical analyses were performed using SPSS version 24.0 (SPSS, Chicago, IL, USA). The normality of the data distribution was assessed by the Kolmogorov–Smirnov test. Continuous data are expressed as mean ± standard deviation (SD) and were analyzed by independent sample *t*-test. Nonnormally distributed data are expressed by median (min–max) and were analyzed by the Mann–Whitney *U* test. Categorical variables are presented as numbers (*n*) with percentages (%) and were analyzed by chi-square test. Probabilities below 0.05 were accepted as significant.

## 3. Results

Between January 2009 and July 2020, 304 patients were diagnosed with PHPT in our hospital including 217 female and 87 male patients. Of the 304 patients, 123 were asymptomatic PHPT patients and 181 were symptomatic PHPT patients. Among the asymptomatic PHPT patients, 65.85% were initially identified by the parathyroid nodules incidentally discovered by neck ultrasound, while 34.15% were identified by increased serum calcium level. Great differences were found between asymptomatic and symptomatic PHPT patients in most of the laboratory parameters (Table 1). Specifically, the asymptomatic PHPT patients showed significantly lower serum calcium, serum chlorine, ALP, PTH, and bone turnover markers (*p* < 0.05), as well as higher serum phosphate, magnesium, potassium, and serum 25 (OH)D level when compared with symptomatic PHPT patients (*p* < 0.05).

Concurrent thyroid nodules were found in 187 patients (61.51%) during ultrasound examination: 87 asymptomatic PHPT cases and 100 symptomatic PHPT cases. The prevalence of thyroid nodules was higher in the asymptomatic PHPT patients than in the symptomatic PHPT patients (70.73% versus 55.24%, *p* < 0.05).

A total of 276 PHPT patients underwent parathyroidectomy, including 168 symptomatic PHPT patients and 108 asymptomatic PHPT patients who met the surgery criteria of the guidelines. 87 of the 276 patients underwent simultaneous thyroid and parathyroid surgery: 49 asymptomatic PHPT cases and 38 symptomatic PHPT cases. Simultaneous thyroid and parathyroid surgery was conducted in the following situation: (1) PTC was confirmed by preoperative FNA (*N* = 7); (2) for the sonographically suspicious thyroid nodules which were not suitable for FNA, the necessity of thyroidectomy was carefully evaluated by experienced surgeons based on the sonographic pattern and clinical manifestations (*N* = 71); (3) the enlarged thyroid gland compressed the trachea (*N* = 8); and (4) potential malignancy of thyroid was found by the surgeon intraoperatively (e.g., abnormal texture of thyroid tissue, *N* = 1). The postoperative histopathology assessment showed nodular goiter in 54 cases, PTC in 26 cases, lymphocytic thyroiditis in 3 cases, and thyroid adenoma in 4 cases; and, for the remaining 100 patients, no FNA biopsy or thyroid surgery was performed due to the limited diameter without high suspicion.

A total of 26 PHPT patients were diagnosed with PTC: 19 asymptomatic PHPT patients and 7 symptomatic PHPT patients. For the 14 asymptomatic PHPT patients who were identified by the parathyroid nodules, the potential malignant thyroid nodules were found at the same time by ultrasound examination; and, for the other 5 asymptomatic PHPT patients who were identified by serum calcium examination and 7 symptomatic PHPT patients, the potential malignant thyroid nodules were detected during the preoperative examination of PHPT. FNA was performed in 7 patients and PTC was confirmed before surgery. For the remaining 19 patients, FNA was not performed due to the limited tumor diameter or admission before July 2016. None of the patients had a history of head or neck radiotherapy. Compared with the PHPT patients with benign thyroid lesion, lower levels of PTH and serum calcium were found in the PHPT patients with PTC (*p* < 0.05). No statistically significant differences were found in the serum phosphate, magnesium, potassium, sodium, chlorine, ALP, and 25 (OH)D results (*p* > 0.05) (Table 2).

Comparison of asymptomatic and symptomatic PHPT patients showed remarkable differences in clinicopathological characteristics of PTC. In particular, PTC was found in 15.44% of the asymptomatic PHPT patients, which was much higher than the percentage in symptomatic PHPT patients (3.87%, *p* < 0.05). All cases of PTC among symptomatic PHPT patients were classic PTC, while 10.53% of the PTC cases in asymptomatic PHPT patients were tall cells variant. All the tumors in symptomatic PHPT patients were of the micropapillary type and limited to the thyroid, while 68.42% of PTC cases in asymptomatic patients showed microscopic extrathyroidal extension. Metastases to lymph nodes in the cervical central area were only observed in asymptomatic PHPT patients too. Multifocality was another feature of the PTC in asymptomatic PHPT patients. Namely, seven patients (36.84%) with asymptomatic PHPT showed more than one focus, whereas only one patient (14.29%) was found among symptomatic PHPT group (Table 3).

## 4. Discussion

Thyroid nodule is a common disease which is often concomitant with PHPT, partly due to the accidental finding in the routine cervical ultrasound examination. According to the former studies, 50–73.3% of PHPT patients had concomitant thyroid nodules, which was consistent with our finding (61.51%) [10, 11]. The relationship between thyroid nodules and asymptomatic PHPT seems even closer. Asymptomatic PHPT was more common in the patients with a history of thyroid abnormalities than in symptomatic PHPT [12]. In the present study, we found that the prevalence of thyroid nodules was higher in the asymptomatic patients than in symptomatic patients, partly caused by the fact that 65.85% of the asymptomatic PHPT patients in this cohort were identified through ultrasound examination, which would facilitate the discovery of both thyroid nodules and enlarged parathyroid. Moreover, the higher PTC prevalence in the asymptomatic PHPT patients in this inpatient cohort might be also caused by the selection bias, since patients with potential malignant thyroid lesions had higher possibility of hospitalization.

PTC is the most common type of the malignant thyroid lesions synchronous with PHPT [13–15]. Ogburn and Black were the first to report the concomitant PTC in PHPT patients in 1956 [16]. Thus far, more than 500 cases of concomitant PTC in PHPT patients have been reported. The prevalence of PTC in PHPT patients varies from 0.9% to 18.2% due to different variables including country, race, and age [15, 17]. The incidence of PTC in Chinese PHPT patients is still unclear. In 2007, Zheng et al. were the first to report 1 case of PTC among 22 PHPT patients who had synchronous thyroid and parathyroid surgery [18]. Xue et al. found 12 PTC cases among 155 surgically treated PHPT patients in 2016 [19]. More recently, Yu et al. have reported that the prevalence of PTC in thyroidectomized PHPT patients was 43.14% (22/51) in 2019. To our knowledge, our study is the largest cohort reporting features of PTC in PHPT patients in China with 26 PTC cases in 304 PHPT patients.

Despite the high incidence of PTC in PHPT patients, the association between sporadic PHPT and PTC is still controversial. Previous studies demonstrated that most of the PTC foci in PHPT patients were papillary microcarcinoma (PMC), which was consistent with our finding [15, 20]. When compared with patients of PTC alone, the concomitant PTC in PHPT patients showed significantly smaller tumor diameters, leading some researchers to believe that the PTC was overdiagnosed in PHPT patients [7, 21]. Preoperative imaging for diagnosis and localization of parathyroid adenomas greatly increased the detection of these indolent tumors coexisting with PHPT [22]. However, some recent studies showed a higher invasive ability of PTC in PHPT patients, indicating the promoting effect of PHPT in PTC development [7, 23–26]. In our study, a great difference in the invasive ability was found between the asymptomatic and symptomatic PHPT patients. In symptomatic PHPT patients, all PTC cases were PMC limited in the thyroid. However, among the concomitant PTC in asymptomatic PHPT patients, 68.42% of the lesions showed microscopic extraglandular invasion, which was much higher than that in the symptomatic PHPT group and that in the general population [27]. So the PTC in symptomatic PHPT patients might be overdiagnosed; however, in asymptomatic PHPT patients, a higher invasive ability was observed in the concomitant PTC.

According to previous studies, the potential mechanisms for high incidence of PTC in PHPT patients included shared embryological origin and genes and high level of PTH and 1,25-hydroxy vitamin D, as well as hypercalcemia [26, 28]. On the one hand, a high level of PTH and 1,25-hydroxy vitamin D could produce an inhibitory effect on various parameters of the immune system and favor cancerogenesis [29–31]. On the other hand, increased mitotic activity induced by hypercalcemia and elevated concentrations of angiogenic growth factors and fibroblast growth factor induced by PTH could stimulate tumor growth [32–34]. These reasons could explain the higher incidence of PTC in PHPT patients than in general population [22]; however, they were inadequate to explain the stronger invasive ability of PTC in asymptomatic PHPT patients whose serum calcium and PTH were lower as we observed; and, for the 1,25-hydroxy vitamin D, although we could not test it in our hospital, previous study showed that the 1,25-hydroxy vitamin D level was lower in asymptomatic PHPT patients when compared to symptomatic PHPT patients [35]. Moreover, both previous studies and the present study showed a lower PTH and serum calcium level in the PHPT patients with concomitant PTC when compared with PHPT patients with benign thyroid lesion [19, 36]. We therefore hypothesized that elevated PTH and calcium could promote the development of PTC; however, this effect may not be dose-dependent.

Asymptomatic PHPT is a milder presentation of PHPT. Due to the absence of typical symptoms, it is difficult to determine the duration of the disease. Previous studies found that, without intervention, 73% and 63% of asymptomatic PHPT patients remained stable without any new complications during a follow-up of 10 years and 15 years, respectively [37, 38]. In this context, asymptomatic PHPT patients might suffer from the slightly elevated serum calcium and PTH level for a long time until diagnosis by an accidental calcium examination or neck ultrasound, especially in developing countries where blood calcium and ultrasound are not yet routinely carried out. Neck ultrasound has been carried out during annual physical examination in our hospital only in the recent five years, and blood calcium screening was only routinely carried out in the hospitalized patients, so the disease duration of asymptomatic PHPT in our cohort might be even longer. Therefore, we assumed that the duration, rather than the degree of the elevated serum calcium and PTH, was the potential risk factor for PTC in PHPT patients.

Surgery is the only potential curative option for patients with PHPT. Despite meeting biochemical criteria for PHPT diagnosis, asymptomatic PHPT patients often do not meet criteria for surgery recommended by guidelines of asymptomatic PHPT [39]. However, according to the American Association of Endocrine Surgeons guidelines, thyroid resection should be performed at the same time of parathyroidectomy in PHPT patients with concomitant thyroid disease that required resection [40]. The higher rate of microscopic extrathyroidal invasion of PTC we found in asymptomatic PHPT patients provided new support data for this recommendation. Moreover, the concomitant thyroid disease laid great influence on the surgical approach of the PHPT patients and concurrent thyroidectomy, and parathyroidectomy would cause longer surgical time and length of stay in hospital [41]. FNA is the most accurate and cost-effective method for evaluating thyroid nodules [42], so it is of great importance to confirm thyroid malignancy by FNA before the parathyroidectomy. This study had certain limitations. First, our results are observational. We found a higher rate of microscopic extrathyroidal invasion of PTC in asymptomatic PHPT patients; however, the underlying mechanism of such invasiveness was still unclear and further studies are needed. Also, we performed a retrospective single-institution study and all the patients in the cohort were hospitalized. Although we included both surgical and nonsurgical patients, the number of asymptomatic PHPT patients might still be underestimated, which would lead to biased representation of the prevalence of both PTC and thyroid nodules in asymptomatic PHPT. Therefore, multicenter studies including clinic and community populations are needed. Furthermore, most of the thyroid nodules graded TIRADS 1–3 were not surgically treated, since previous studies proved that the malignancy rate was only 2–4% in these nodules [9]. However, it cannot rule out the existence of potential thyroid carcinoma, and long-term follow-up of these patients is needed.

## 5. Conclusions

In this largest cohort of Chinese patients with coexisting PTC and PHPT, we found that the prevalence of PTC in thyroidectomized PHPT patients was 29.89%. We demonstrated differences in pathological features of PTC between asymptomatic and symptomatic PHPT patients for the first time. Although asymptomatic PHPT patients showed milder laboratory abnormalities, a higher rate of microscopic extrathyroidal invasion in the concomitant PTC was observed. So we suggested that FNA should be performed in the asymptomatic PHPT patients with suspicious thyroid nodules; also the concurrent thyroidectomy and parathyroidectomy were necessary when the diagnosis of PTC was confirmed.

## Figures and Tables

**Table 1:** Comparison of clinical feature and laboratory values between asymptomatic PHPT patients and symptomatic PHPT patients.

	Total PHPT (*N* = 304)	Asymptomatic PHPT (*N* = 123)	Symptomatic PHPT (*N* = 181)	*p* value
Age	53.27 ± 13.54	53.68 ± 12.53	53.00 ± 14.62	0.67
Female/male ratio	2.49 : 1	3.24 : 1	2.12 : 1	0.12
Calcium (mmol/L)	2.93 (2.62–4.97)	2.77 (2.62–3.46)	3.12 (2.66–4.97)	<0.001
Albumin-corrected calcium (mmol/L)	2.87 (2.51–5.04)	2.68 (2.51–3.81)	3.11 (2.56–5.04)	<0.001
Phosphate (mmol/L)	0.75 ± 0.21	0.83 ± 0.16	0.69 ± 0.22	<0.001
Magnesium (mmol/L)	0.88 (0.4–1.11)	0.92 (0.7–1.06)	0.84 (0.4–1.11)	<0.001
Potassium (mmol/L)	4.18 (2.6–5.44)	4.22 (3.21–5.44)	4.13 (2.6–5.27)	<0.001
Sodium, mmol/L)	141 (131–151)	141 (135–147)	141 (131–151)	0.93
Chlorine (mmol/L)	107 (94–119)	107 (96–116)	108 (94–119)	0.02
ALP (U/L)	125 (41–2644)	101 (44–405)	152 (41–2644)	<0.001
PTH (pg/mL)	303.95 (30.63–4595)	170.10 (30.63–1666)	552.5 (34.54–4595)	<0.001
25 (OH)D (ng/mL)	11.35 (3–32.34)	14.20 (3–32.34)	9.30 (3–27.75)	0.02
N-BGP (ng/mL)	61.10 (14.21–300)	37.76 (14.21–188.30)	92.76 (19.3–300)	<0.001
*β*-CTx (ng/mL)	1.18 (0.19–6.00)	0.90 (0.29–3.61)	1.48 (0.19–6.00)	<0.001
TP1NP (ng/mL)	108.40 (9.55–1200)	80.98 (23.13–154.80)	125.85 (9.55–1200)	<0.001

PHPT, primary hyperparathyroidism; ALP, alkaline phosphatase; PTH, parathyroid hormone; 25 (OH)D, 25-hydroxyvitamin D; N-BGP, N-bone Gla-protein; *β*-CTx, *β*-isomerized C-terminal telopeptides; TP1NP, total procollagen type 1 amino-terminal propeptide.

**Table 2:** Comparison of serum laboratory markers between PHPT patients with PTC or benign thyroid lesion.

	PHPT with PTC, *N* = 26	PHPT with benign thyroid lesion, *N* = 161	*p* value
Calcium (mmol/L)	2.74 (2.68–4.97)	2.93 (2.62–4.78)	0.02
Phosphate (mmol/L)	0.81 ± 0.20	0.75 ± 0.21	0.16
Magnesium (mmol/L)	0.89 (0.56–1.06)	0.89 (0.4–1.11)	0.56
Potassium (mmol/L)	4.19 ± 0.40	4.16 ± 0.44	0.74
Sodium (mmol/L)	143 (131–145)	141 (132–151)	0.11
Chlorine (mmol/L)	108.50 (102–114)	107 (96–117)	0.45
ALP (U/L)	108.50 (56–734)	133.50 (44–2540)	0.21
PTH (pg/mL)	160.55 (30.63–3576)	305.80 (65.88–4278)	0.02
25 (OH)D (ng/mL)	12.89 ± 7.33	10.90 ± 6.02	0.32

PHPT, primary hyperparathyroidism; PTC, papillary thyroid carcinoma; ALP, alkaline phosphatase; PTH, parathyroid hormone; 25 (OH)D, 25-hydroxyvitamin D.

**Table 3:** Pathological characteristics of papillary thyroid carcinoma in asymptomatic PHPT and symptomatic PHPT patients.

	Asymptomatic PHPT and PTC (*N* = 19)		Symptomatic PHPT and PTC (*N* = 7)		*p* value
	Number	Percent (%)	Number	Percent (%)	
*T (tumor) classification*					
T1a	15	78.95	7	100%	0.19
T1b	4	21.05	0	0	

*N (lymph node) classification*					
Nx	3	15.79	2	28.57%	0.39
N0	13	68.42	5	71.43%	
N1	3	15.79	0	0	

Mean tumor size, cm	0.64 ± 0.35		0.60 ± 0.33		0.74
Multifocality	7	36.84	1	14.28%	0.24
Microscopic extrathyroidal invasion	13	68.42	0	0	<0.001

PHPT, primary hyperparathyroidism; PTC, papillary thyroid carcinoma.

## Data Availability

The data used to support the findings of this study are available from the corresponding author upon request.

## References

[1] Adami S., Marcocci C., Gatti D. (2002). Epidemiology of primary hyperparathyroidism in Europe. *Journal of Bone and Mineral Research: The Official Journal of the American Society for Bone and Mineral Research*.

[2] Lo C.-Y. (2004). Surgical treatment for primary hyperparathyroidism in Hong Kong: changes in clinical pattern over 3 decades. *Archives of Surgery*.

[3] Liu J.-M., Cusano N. E., Silva B. C. (2013). Primary hyperparathyroidism: a tale of two cities revisited-New York and shanghai. *Bone Research*.

[4] Zhao L., Pang P., Zang L. (2019). Features and trends of thyroid cancer in patients with thyroidectomies in Beijing, China between 1994 and 2015: a retrospective study. *BMJ Open*.

[5] Du L., Wang Y., Sun X. (2018). Thyroid cancer: trends in incidence, mortality and clinical-pathological patterns in Zhejiang Province, Southeast China. *BMC Cancer*.

[6] Zhong Z., Yuan J., Chen X. (2020). The clinicopathological features of papillary thyroid carcinoma patients with positive hepatitis B surface antigen. *Oncology Research and Treatment*.

[7] Çetin K., Sıkar H. E., Temizkan Ş. (2019). Does primary hyperparathyroidism have an association with thyroid papillary cancer? A retrospective cohort study. *World Journal of Surgery*.

[8] Walker M. D., Silverberg S. J. (2018). Primary hyperparathyroidism. *Nature Reviews Endocrinology*.

[9] Horvath E., Majlis S., Rossi R. (2009). An ultrasonogram reporting system for thyroid nodules stratifying cancer risk for clinical management. *The Journal of Clinical Endocrinology & Metabolism*.

[10] Morita S. Y., Somervell H., Umbricht C. B., Dackiw A. P. B., Zeiger M. A. (2008). Evaluation for concomitant thyroid nodules and primary hyperparathyroidism in patients undergoing parathyroidectomy or thyroidectomy. *Surgery*.

[11] Phillips D. J., Kutler D. I., Kuhel W. I. (2014). Incidental thyroid nodules in patients with primary hyperparathyroidism. *Head & Neck*.

[12] Latina A., Castellano E., Cesario F., Boriano A., Attanasio R., Borretta G. (2018). Unknown and already known thyroid abnormalities in primary hyperparathyroidism. *Endocrine Practice*.

[13] Celik M., Guldiken S., Ayturk S. (2017). Benign and malignant thyroid gland diseases in the patients with primary hyperparathyroidism. *International Journal of Applied and Basic Medical Research*.

[14] Tsai C.-Y., Chen S.-T., Hsueh C., Lin Y.-S., Lin J.-D. (2020). Long-term therapeutic outcomes of papillary thyroid carcinoma with concomitant hyperparathyroidism: a single center case-control study. *Biomedical Journal*.

[15] Preda C., Branisteanu D., Armasu I. (2019). Coexistent papillary thyroid carcinoma diagnosed in surgically treated patients for primary versus secondary hyperparathyroidism: same incidence, different characteristics. *BMC Surgery*.

[16] Ogburn P. L., Black B. M. (1956). Primary hyperparathyroidism and papillary adenocarcinoma of the thyroid; report of four cases. *Proceedings of the Staff Meetings. Mayo Clinic*.

[17] Burmeister L. A., Sandberg M., Carty S. E., Watson C. G. (1997). Thyroid carcinoma found at parathyroidectomy: association with primary, secondary, and tertiary hyperparathyroidism. *Cancer*.

[18] Zheng Y.-X., Xu S.-M., Wang P., Chen L. (2007). Preoperative localization and minimally invasive management of primary hyperparathyroidism concomitant with thyroid disease. *Journal of Zhejiang University Science B*.

[19] Xue Y., Ye Z.-Q., Zhou H.-W., Shi B.-M., Yi X.-H., Zhang K.-Q. (2016). Serum calcium and risk of nonmedullary thyroid cancer in patients with primary hyperparathyroidism. *Medical Science Monitor*.

[20] Kutluturk K., Otan E., Yagci M. A., Usta S., Aydin C., Unal B. (2014). Thyroid pathologies accompanying primary hyperparathyroidism: a high rate of papillary thyroid microcarcinoma. *Turkish Journal of Surgery*.

[21] Vaccarella S., Franceschi S., Bray F., Wild C. P., Plummer M., Dal Maso L. (2016). Worldwide thyroid-cancer epidemic? The increasing impact of overdiagnosis. *New England Journal of Medicine*.

[22] Cinamon U., Turcotte R. E. (2006). Primary hyperparathyroidism and malignancy: “Studies by nature. *Bone*.

[23] Lever E. G., Refetoff S., Straus F. H., Nguyen M., Kaplan E. L. (1983). Coexisting thyroid and parathyroid disease--are they related?. *Surgery*.

[24] Kösem M., Algün E., Kotan Ç., Harman M., Öztürk M. (2004). Coexistent thyroid pathologies and high rate of papillary cancer in patients with primary hyperparathyroidism: controversies about minimal invasive parathyroid surgery. *Acta Chirurgica Belgica*.

[25] Bentrem D. J., Angelos P., Talamonti M. S., Nayar R. (2002). Is preoperative investigation of the thyroid justified in patients undergoing parathyroidectomy for hyperparathyroidism?. *Thyroid*.

[26] Beebeejaun M., Chinnasamy E., Wilson P., Sharma A., Beharry N., Bano G. (2017). Papillary carcinoma of the thyroid in patients with primary hyperparathyroidism: is there a link?. *Medical Hypotheses*.

[27] Dideban S., Abdollahi A., Meysamie A., Sedghi S., Shahriari M. (2016). Thyroid papillary microcarcinoma: etiology, clinical manifestations, diagnosis, follow-up, histopathology and prognosis. *Iranian Journal of Pathology*.

[28] Roskies M., Dolev Y., Caglar D (2012). Vitamin D deficiency as a potentially modifiable risk factor for thyroid cancer. *Journal of otolaryngology-head & neck surgery*.

[29] Geara A. S., Castellanos M. R., Bassil C. (2010). Effects of parathyroid hormone on immune function. *Clinical and Developmental Immunology*.

[30] Tzanno-Martins C., Futata E., Jorgetti V., Duarte A. J. (2000). Immune response in hemodialysis patients: is there any difference when low and high iPTH levels are compared?. *Clinical Nephrology*.

[31] El-Fakhri N., McDevitt H., Shaikh M. G., Halsey C., Ahmed S. F. (2014). Vitamin D and its effects on glucose homeostasis, cardiovascular function and immune function. *Hormone Research in Paediatrics*.

[32] Jiang L., Zhang W., Wei L. (2018). Early effects of parathyroid hormone on vascularized bone regeneration and implant osseointegration in aged rats. *Biomaterials*.

[33] Veselý D., Astl J., Matucha P., Sterzl I., Betka J. (2003). Serum levels of angiogenic growth factors in patients with thyroid gland tumors and parathyroid adenoma. *Neuro Endocrinology Letters*.

[34] Yazici P., Mihmanli M., Bozdag E., Aygun N., Uludag M. (2015). Incidental finding of papillary thyroid carcinoma in the patients with primary hyperparathyroidism. *The Eurasian Journal of Medicine*.

[35] Clarke B. L. (2019). Asymptomatic primary hyperparathyroidism. *Parathyroid Disorders*.

[36] dell’Erba L., Baldari S., Borsato N. (2001). Retrospective analysis of the association of nodular goiter with primary and secondary hyperparathyroidism. *European Journal of Endocrinology*.

[37] Silverberg S. J., Shane E., Jacobs T. P., Siris E., Bilezikian J. P. (1999). A 10-year prospective study of primary hyperparathyroidism with or without parathyroid surgery. *New England Journal of Medicine*.

[38] Rubin M. R., Bilezikian J. P., McMahon D. J. (2008). The natural history of primary hyperparathyroidism with or without parathyroid surgery after 15 years. *The Journal of Clinical Endocrinology & Metabolism*.

[39] Bilezikian J. P., Brandi M. L., Eastell R. (2014). Guidelines for the management of asymptomatic primary hyperparathyroidism: summary statement from the Fourth International Workshop. *The Journal of Clinical Endocrinology & Metabolism*.

[40] Wilhelm S. M., Wang T. S., Ruan D. T. (2016). The American association of endocrine surgeons guidelines for definitive management of primary hyperparathyroidism. *JAMA Surgery*.

[41] Castellano E., Benso P., Attanasio R. (2020). Surgical approach to primary hyperparathyroidism in patients with concomitant thyroid diseases: a retrospective single center study. *International Journal of Endocrinology*.

[42] Patel K. N., Yip L., Lubitz C. C. (2020). The American association of endocrine surgeons guidelines for the definitive surgical management of thyroid disease in adults. *Annals of Surgery*.

